# Conventional Versus Underwater Endoscopic Mucosal Resection for Superficial Non-Ampullary Duodenal Epithelial Tumors ≤ 20 mm: Study Protocol for a Multicenter Randomized Controlled Trial (D-CURE Trial)

**DOI:** 10.3390/mps9010030

**Published:** 2026-02-23

**Authors:** Masao Yoshida, Waku Hatta, Tomohiro Nakamura, Naoki Nakaya, Satoki Shichijo, Yasuyuki Tanaka, Hiromitsu Kanzaki, Kingo Hirasawa, Ichiro Oda, Takashi Hirose, Motohiko Kato, Kohei Takizawa, Yosuke Toya, Takuto Hikichi, Hiroaki Sawai, Naohiro Yoshida, Osamu Dohi, Atsushi Masamune, Seiichiro Abe, Tomonori Yano

**Affiliations:** 1 Division of Endoscopy, Shizuoka Cancer Center, Shizuoka 411-8777, Japan; 2 Division of Gastroenterology, Tohoku University Graduate School of Medicine, Miyagi 980-8574, Japan; 3 Faculty of Data Science, Kyoto Women’s University, Kyoto 605-8501, Japan; 4 Department of Preventive Medicine and Epidemiology, Tohoku Medical Megabank Organization, Tohoku University, Miyagi 980-0872, Japan; 5 Department of Gastrointestinal Oncology, Osaka International Cancer Institute, Osaka 541-8567, Japan; 6 Department of Gastroenterology and Hepatology, Kyoto Katsura Hospital, Kyoto 615-8256, Japan; 7 Department of Internal Medicine, Tsuyama Chuo Hospital, Okayama 708-0841, Japan; 8 Division of Endoscopy, Yokohama City University Medical Center, Kanagawa 232-0024, Japan; 9 Department of Internal Medicine, Kawasaki Rinko General Hospital, Kanagawa 210-0804, Japan; 10 Department of Gastroenterology and Hepatology, Nagoya University Graduate School of Medicine, Aichi 466-8550, Japan; 11 Center for Diagnostic and Therapeutic Endoscopy, Keio University School of Medicine, Tokyo 160-8582, Japan; 12 Department of Endoscopy, Kanagawa Cancer Center, Kanagawa 241-8515, Japan; 13 Division of Gastroenterology, Department of Internal Medicine, Iwate Medical University, Iwate 020-8505, Japan; ytoya@iwate-med.ac.jp; 14 Department of Endoscopy, Fukushima Medical University Hospital, Fukushima 960-1295, Japan; 15 Department of Gastroenterology, Takatsuki General Hospital, Osaka 569-1192, Japan; 16 Department of Gastroenterology, Ishikawa Prefectural Central Hospital, Ishikawa 920-8530, Japan; 17 Molecular Gastroenterology and Hepatology, Graduate School of Medical Science, Kyoto Prefectural University of Medicine, Kyoto 602-8566, Japan; 18 Endoscopy Division, National Cancer Center Hospital, Tokyo 104-0045, Japan; 19 Department of Gastroenterology and Endoscopy, National Cancer Center Hospital East, Chiba 277-8577, Japan

**Keywords:** conventional endoscopic mucosal resection, underwater endoscopic mucosal resection, sporadic non-ampullary duodenal epithelial tumors, non-inferiority randomized controlled trial, recurrence-free survival rate

## Abstract

**Background:** Underwater endoscopic mucosal resection (UEMR) is a relatively new treatment method for sporadic non-ampullary duodenal epithelial tumors (SNADETs), and its usefulness has been reported for SNADETs ≤ 20 mm. However, its effectiveness and safety compared with conventional endoscopic mucosal resection (CEMR) remain controversial. This study aims to assess the treatment outcomes and evaluate the beneficial effects and safety of UEMR for SNADETs ≤ 20 mm. **Methods:** This is an open-label, multicenter collaborative, non-inferiority randomized controlled trial with two parallel groups conducted across 40 institutions in Japan. The study subjects will be patients with SNADETs ≤ 20 mm. A total of 320 patients will be randomized to either the CEMR or UEMR group in a 1:1 allocation ratio. The primary endpoint is the 1-year recurrence-free survival rate, defined as the number of cases with no recurrence of SNADET or death from any cause within one year of EMR. The secondary endpoints include the *en bloc* resection rate, histological complete resection rate, adverse events, technical success rate, total procedure time, resection time, mucosal closure time, complete mucosal closure rate, complete mucosal closure rate with standard clips, 1-year duodenum preservation survival rate, and device cost. **Discussion:** This multicenter, open-label, randomized controlled trial (RCT) with 320 subjects aims to determine whether the 1-year recurrence-free survival rate of underwater endoscopic resection is not inferior to that of conventional endoscopic mucosal resection for SNADETs ≤ 20 mm. If the efficacy and safety of UEMR are proven in this RCT, it is expected to be recognized as the standard treatment for SNADETs ≤ 20 mm. Owing to the absence of submucosal injection, UEMR is a simpler and more cost-effective technique compared to CEMR and is anticipated to become the primary method of EMR.

## 1. Background

Sporadic non-ampullary duodenal epithelial tumors (SNADETs), which seldom metastasize to lymph nodes, are generally considered more suitable for less-invasive endoscopic resection (ER) than for surgery [1]. However, there are challenges associated with ER for SNADETs owing to the specific anatomical features of the duodenum, such as thin walls, exposure to bile and pancreatic juices, and poor endoscopic maneuverability. Although conventional endoscopic mucosal resection (CEMR) is more widely adopted owing to its simplicity and safety, complication rates are higher in the duodenal ER than in the ER of other digestive tracts owing to these factors [2].

There is a lack of comparative studies between CEMR and other ER methods, leading to the absence of established standard treatments for SNADETs ≤ 20 mm [3]. Notably, the American Society for Gastrointestinal Endoscopy guidelines do not specifically mention the standard treatments [4]. In contrast, the European Society of Gastrointestinal Endoscopy guidelines established CEMR as the standard treatment, irrespective of the tumor size [5]. Because *en bloc* resection of SNADETs > 20 mm is often difficult using EMR, endoscopic submucosal dissection (ESD) is performed in Japan. Before starting this trial, the community reached a consensus to set CEMR as the standard ER for SNADETs ≤ 20 mm. However, preoperative biopsy scars and the presence of Brunner’s glands in the submucosal layer can cause inadequate mucosal elevation through submucosal injection in CEMR, leading to piecemeal resection or conversion to ESD [6]. Additionally, piecemeal resection increases the risk of recurrence, which may require surgical intervention if recurrence occurs, indicating the need for improvement in CEMR [7,8,9,10].

Recently, the feasibility of underwater EMR (UEMR) as a method to overcome the difficulties associated with CEMR has been reported [11]. UEMR is an ER technique that uses the phenomenon of lesions floating toward the lumen by filling the duodenal lumen with water or saline, resembling the effect of a submucosal injection. Because UEMR does not require submucosal injection, it can address the difficulty of resection owing to insufficient mucosal elevation, a common issue in CEMR. Additionally, UEMR offers the advantage of stable snaring without snare slippage owing to insufficient mucosal elevation, even in flat lesions. Furthermore, owing to the villous mucosa of the duodenum and thin proper muscle, submucosal injection is relatively challenging compared with injection into other gastrointestinal tracts. The performance of UEMR for SNADETs ≤ 20 mm has been evaluated in a multicenter study, where the results indicated an *en bloc* resection rate of 89.9%, a histological complete *en bloc* resection rate of 66.9%, an intraoperative perforation rate of 0%, a postoperative bleeding rate of 1.2%, a delayed perforation rate of 0%, and a one-year recurrence-free survival rate of 97.2%, suggesting its safety and efficacy [12]. The reported histologically complete *en bloc* resection rate is lower for UEMR than for CEMR [2,13,14,15,16], which may be associated with local recurrence after UEMR. However, it is important to note that the single-arm trial was subject to selection bias, and the treatment outcomes of the CEMR used as the historical control may differ from the current data. Additionally, it is unclear whether the trial data can be externally validated [3]. Therefore, it is necessary to demonstrate the non-inferiority of the local recurrence rate of UEMR using direct comparison with CEMR.

The D-CURE trial aims to assess the treatment outcomes and evaluate the beneficial effects and safety of UEMR for SNADETs ≤ 20 mm.

## 2. Methods and Analysis

### 2.1. Study Settings

**Figure 1:**
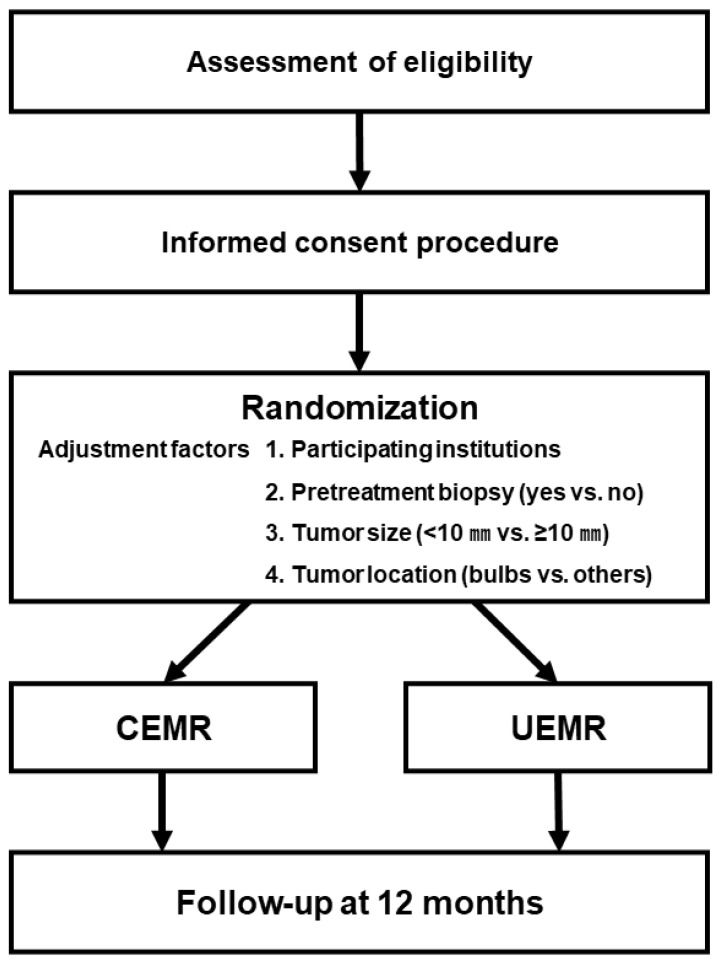
Flowchart of the D-CURE trial. CEMR, conventional endoscopic mucosal resection; UEMR, underwater endoscopic mucosal resection.

**Figure 2:**
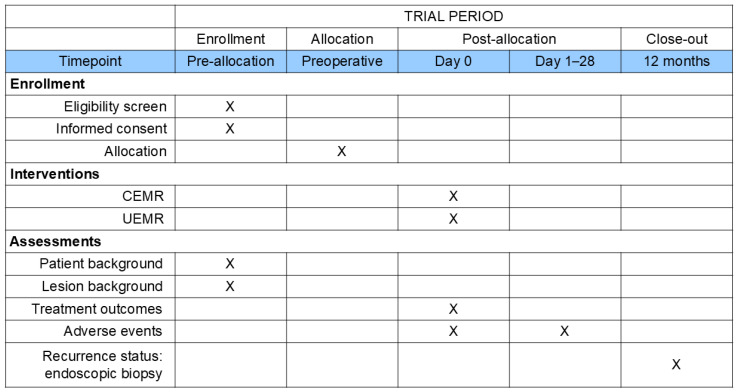
Time schedule for the D-CURE trial. CEMR, conventional endoscopic mucosal resection; UEMR, underwater endoscopic mucosal resection.

The D-CURE trial is an open-label, multicenter, collaborative, non-inferiority randomized controlled trial (RCT) with two parallel groups, conducted across 40 institutions in Japan. The control arm will include patients who undergo CEMR, whereas the study arm will include patients who undergo UEMR. Figure 1 shows the flowchart of the D-CURE trial. The trial protocol was developed in accordance with the Standard Protocol Items: Recommendations for Interventional Trials (SPIRIT) guidelines [17]. The SPIRIT flow diagram is shown in Figure 2.

### 2.2. Approvals

The study protocol was approved by the certified review board of Tohoku University (No. 34158; 22 November 2023). The trial was registered in the University Hospital Medical Information Network (UMIN) Clinical Trials Registry (UMIN000052778; 17 November 2023).

### 2.3. Patient Selection

Patients who meet all the inclusion criteria and none of the exclusion criteria will be considered eligible for enrollment.

#### 2.3.1. Inclusion Criteria

1. Endoscopically diagnosed adenoma, adenocarcinoma, or histologically confirmed adenoma or adenocarcinoma through preoperative biopsy.
2. The lesion has a longitudinal diameter of 20 mm or less.
3. The estimated tumor depth is limited to the mucosa.
4. The lesion is located in the duodenal bulb, descending part, or horizontal part, without invasion into the major or minor papilla.
5. The lesion is non-pedunculated.
6. Scheduled for endoscopic treatment for a single lesion.
7. No history of gastrectomy or duodenectomy.
8. Age at the time of registration ranges from 18 years to 85 years.
9. Eastern Cooperative Oncology Group Performance Status is 0, 1, or 2.
10. Has received adequate explanation regarding trial participation and has provided written consent.

#### 2.3.2. Exclusion Criteria

1. Diagnosed with familial adenomatous polyposis (FAP).
2. Presents with systemic infections requiring systemic treatment.
3. Pregnant or breastfeeding women.
4. Psychiatric disorders, psychiatric symptoms, or dementia that would hinder participation in the trial.
5. Complicated by unstable angina (occurrence within the last three weeks or exacerbation of angina) or a history of myocardial infarction within the past six months.
6. Patients taking multiple antithrombotic drugs for whom endoscopy is recommended to be postponed according to the Japanese guidelines for gastrointestinal endoscopic treatment of patients receiving antithrombotic agents [18,19].
7. Complicated with respiratory diseases requiring continuous oxygen therapy.
8. Patients with a prognosis of less than 1 year.
9. Other patients deemed inappropriate by the attending trial physician.

### 2.4. Informed Consent Procedure

The patients will be screened for eligibility by endoscopists at each institution based on the abovementioned criteria. The principal investigator or the physician in charge will obtain free and voluntary written consent from the patients after providing a full explanation of the research using explanatory documents (Supplemental Material File). Written informed consent will be obtained from all patients who wish to participate in this study. They will also be informed that they can withdraw their consent at any time.

### 2.5. Randomization

A randomization process will be conducted using a central web-based system to assign patients to either the CEMR or UEMR group in a 1:1 allocation ratio. For random allocation, a minimization method will be used, incorporating four allocation adjustment factors that may influence the treatment outcomes of EMR. Allocation concealment will be ensured using a central web-based system, through which treatment assignments will be generated only after patient registration and confirmation of eligibility. Investigators and endoscopists will not have access to the allocation sequence in advance and will be unable to predict upcoming assignments.

#### Randomization Adjustment Factors and Their Selected Reasons for This Selection

1. Institution

In a multicenter collaborative study, variations among enrolled patients and subtle differences in treatment methods between facilities are anticipated to potentially influence EMR outcomes.

2. Pretreatment biopsy (presence vs. absence)

Pretreatment biopsies are often performed to confirm histology. However, 24.6% of patients with preoperative biopsies may encounter non-lifting signs after submucosal injection owing to biopsy-induced fibrosis [6]. Therefore, preoperative biopsies can affect the treatment outcomes of EMR.

3. Tumor size (<10 mm vs. ≥10 mm)

As the tumor size increases, CEMR and UEMR show lower *en bloc* resection rates [15]. For lesions measuring 5–9 mm, the *en bloc* resection rates were 95.8% and 93.3% for CEMR and UEMR, respectively. For lesions measuring 10–14 mm, the *en bloc* resection rates were 91.3% and 81.5% for CEMR and UEMR, respectively. For lesions measuring 15–19 mm, the *en bloc* resection rates were 74.4% and 62.0% for CEMR and UEMR, respectively. The maximum tumor diameter in this trial is 20 mm; therefore, a cut-off value of 10 mm was set.

4. Tumor location (bulb vs. other portions)

Gastric-type mucinous lesions are often found in the duodenal bulb, whereas intestinal-type mucinous lesions typically present in the descending part of the duodenum towards the anal side, particularly below the major papilla [20,21,22]. Gastric-type and intestinal-type adenocarcinomas differ in the frequency of submucosal invasion, which likely affects the long-term effectiveness of EMR.

### 2.6. Blinding

Owing to the study design, blinding endoscopists is not feasible. Endoscopists will be aware of the allocated groups at the time of treatment. Patients will also be informed of their assigned groups. Pathological assessment will be performed by pathologists who are blinded to treatment allocation.

### 2.7. Interventions

The protocol treatment will be initiated within 90 days of registration. Each patient with SNADET will be treated using the assigned endoscopic procedure during hospitalization. Only one target lesion will be treated per patient. For CEMR and UEMR in this trial, only physicians who meet the following conditions will be allowed to participate in the treatment as surgeons: (1) CEMR: Experience with CEMR in any organ in over 20 cases and (2) UEMR: Experience with UEMR in any organ in over 20 cases. Intraoperative sedation should follow the guidelines for sedation in endoscopic treatment supervised by the Japan Gastroenterological Endoscopy Society [23]. Treatment under general anesthesia is also permitted. During EMR, the patient’s status should be monitored using blood pressure, electrocardiography, and oxygen saturation.

#### 2.7.1. Standard Treatment Group (CEMR Group)

1. Submucosal injections must be administered with a solution commonly used at each institution. The injection time will be considered as the start time of the CEMR procedure.
2. The lesion, including the surrounding non-tumor mucosa, should be strangled with a commercially available snare of any type or size, chosen at the discretion of each endoscopist. Bipolar snares can be used. However, EMR with the cap technique is not permitted. Although the following settings are recommended for high-frequency devices, adjustments may be made at each facility: Endo-Cut Q mode, effect 2, duration 1, interval 6 for the VIO 300D ERBE; Endo-cut Q, effect 2 for the VIO3. Specimen retrieval should be performed using a retrieval net, grasping forceps, or a snare at the discretion of the endoscopist. Intentional fragmentation is not allowed.

#### 2.7.2. Trial Treatment Group (UEMR Group)

1. After deflating the stomach and duodenum, the duodenal lumen must be filled with saline solution. The start time of saline immersion will be considered the start time of the UEMR procedure. Gel immersion is not permitted in this case.
2. The lesion, including the surrounding non-tumor mucosa, should be snared under saline immersion using a commercially available snare of any type or size, chosen at the discretion of each endoscopist. Bipolar snares can be used. Although the following settings are recommended for high-frequency devices, adjustments may be made at each facility: Endo-Cut Q mode, effect 3, duration 2, interval 4 for VIO 300D ERBE, and Endo-Cut Q, effect 3 for VIO3. Specimen retrieval was performed in the same manner as in CEMR.

#### 2.7.3. Confirmation of Residual Lesions After EMR and Hemostasis

The ulcer bed will be carefully observed after EMR. If any residual lesion is identified, the allocated treatment will be repeated until the lesion is completely resected. For small residual lesions, additional excision using cold snare polypectomy, cold forceps polypectomy, or hot forceps polypectomy is permitted. However, intentional additional cauterization of the residual lesion using a snare or forceps is not permitted. In cases of bleeding, hemostasis will be achieved using clips or cauterization.

#### 2.7.4. Mucosal Closure of EMR Ulcer Bed

Commercially available standard clips with two arms will be used for complete mucosal closure. If closure with a standard clip is challenging, the line-with-clip method or an over-the-scope clip (OTSC) is permitted. If closure is performed using a device other than the standard clip, it is considered a failure of standard clip mucosal closure. The mucosal closure time will be calculated by subtracting the resection time from the total procedural time. Photographs will be captured after the completion of closure and before finishing the procedure.

#### 2.7.5. Rescue Treatment in Case of Incompletion of Allocated Endoscopic Resection Method

If the allocated endoscopic resection method is attempted repeatedly and is not completed within 15 min from the beginning of treatment, the endoscopist may switch to an alternative treatment method, such as CEMR, UEMR, ESD, or surgical resection at their discretion. In this case, the protocol treatment will be discontinued, and the procedure will be considered a failure to achieve completion.

### 2.8. Surveillance

Follow-up esophagogastroduodenoscopy (EGD) will be performed 12 ± 2 months after the EMR, and a biopsy will be obtained from the EMR scar. However, a biopsy may be omitted if the EMR scar is unclear during the follow-up EGD.

### 2.9. Outcomes

#### 2.9.1. Primary Endpoint (For Non-Inferiority)

The primary endpoint is the 1-year recurrence-free survival rate (1-year RFS), defined as the number of cases where no recurrence of SNADETs or death from any cause is observed within one year (12 ± 2 months) after EMR, with all treated cases as the denominator. Cases in which follow-up EGD cannot be performed because of tracking difficulties will be considered non-recurrent.

#### 2.9.2. Secondary Endpoints

The secondary endpoints and their definitions are as follows:

1. *En bloc* resection rate

The *en bloc* resection rate is defined as the proportion of cases in which complete removal of the lesion in a single piece without residual visible tumors is achieved, with the number of treated cases as the denominator. Cases in which resection is performed using methods other than the allocated EMR will be considered failures.

2. Histological complete resection rate

Resected specimens will be appropriately oriented and pinned onto a specimen board, such as a cork, before fixation in formalin. The histological complete resection rate is defined as the proportion of cases in which *en bloc* resection is achieved without histological tumor infiltration at the horizontal and vertical margins in the specimen. The number of treated patients will be the denominator.

3. Adverse events

The adverse event rate is defined as the proportion of patients with one or more expected or unexpected complications observed up to 28 days after EMR, with the treated patients as the denominator. Adverse events will be monitored and recorded as follows:

a. Intraoperative perforation

Intraoperative perforation is defined as a suspected perforation during EMR or the detection of free air on radiography or CT immediately after EMR.

b. Intraoperative bleeding

Massive intraoperative bleeding is defined as bleeding that occurs during EMR and requires interventional radiology or surgery.

c. Delayed bleeding

Delayed bleeding is defined as clinical findings of bleeding (hematemesis, melena, or a decrease in Hb by ≥2 g/dL) observed within 28 days after EMR and confirmed bleeding from the ulcer site after EMR by EGD. Gastric or duodenal blood retention and bleeding from the ulcer site after EMR observed during second-look endoscopy without the above clinical findings are not considered postoperative bleeding.

d. Delayed perforation

Delayed duodenal perforation is defined as perforation not suspected during the procedure and first recognized on the day following the completion of EMR or later.

4. Technical success rate

Technical success is considered when the lesion is completely removed using the allocated EMR method. The number of treated patients is the denominator.

5. Total procedure time

The total procedural time is calculated only for cases of successful resection (per-protocol analysis).

CEMR: Time from the start of submucosal injection to the completion of mucosal closure.

UEMR: Time from the start of saline immersion to the completion of mucosal closure.

6. Resection time

The resection time is calculated only for cases with successful resection (per-protocol analysis).

CEMR: Time from the start of submucosal injection to the completion of lesion resection.

UEMR: Time from the start of saline immersion to the completion of lesion resection.

7. Mucosal closure time

The mucosal closure time is defined as the time obtained by subtracting the resection time from the total procedural time. Closure time is calculated only for cases with successful resection.

8. Complete mucosal closure rate

The complete mucosal closure rate is defined as the proportion of cases in which complete closure is achieved among all treated cases. Complete mucosal closure is considered if the EMR ulcer bed is not visible.

9. Complete mucosal closure rate with standard clips

Complete mucosal closure rate with standard clips is defined as the proportion of patients in whom mucosal closure is completed using only standard clips with two arms without using the clip-line method or OTSC. The number of treated patients is the denominator.

10. One-year duodenum preservation survival rate

The one-year duodenal preservation survival rate is defined as the proportion of patients in whom the duodenum is preserved without undergoing any surgical resection, and those who survive for one year after EMR. Any surgical duodenal resection and cause of death are considered events. If surgical resection occurs, information on the resected specimen will also be collected.

11. Device cost

The total cost is calculated for all treated cases and includes the cost of single-use devices (snare, ESD knife, hemostatic forceps, injection needle, injection fluid, submucosal injection fluid, clips, and OTSC) used during endoscopic resection. Device costs will be calculated based on Japanese reimbursement prices for single-use devices.

### 2.10. Sample Size Calculation

The sample size is calculated based on the primary endpoint, 1-year RFS, to assess the non-inferiority of UEMR to CEMR.

Based on previous studies, the histological complete resection rate of CEMR for SNADET is reported to be 92–100% [2,14,24]. However, these reports are all retrospective studies with relatively short follow-up durations, which could result in a lower 1-year RFS. In this trial, the 1-year RFS in the standard treatment (CEMR) group is set at 97%. Additionally, the non-inferiority margin is set at 5%. This margin was defined as the maximum acceptable absolute excess risk of recurrence at 1 year. Prior studies of endoscopic resection for SNADETs reported very low recurrence rates under standard management. In this low-event-rate setting, an absolute margin is considered clinically interpretable. The prespecified margin of 5% was determined through multidisciplinary clinical consensus, balancing a small allowable increase in recurrence risk against the anticipated procedural advantages of UEMR.

Although the significance level of the trial is typically one-sided at 0.025 [25], the probability of decision-making error in the population with tumors at 0.05 (1 out of 20) is not considered unreasonably high according to clinical trial principles. Considering that a one-sided significance level of 0.05 was accepted in previous studies on rare diseases [12], a one-sided significance level of 0.05 is set for this study. This significance level was selected to maintain statistical feasibility in a rare disease setting with an expected extremely low event rate.

Based on these considerations, assuming a 1-year RFS of 97% for CEMR and expecting UEMR to perform equivalently, with a one-sided α error of 0.05, a non-inferiority margin of 5%, and ensuring 80% statistical power, the required sample size was calculated to be 288 cases for conducting a non-inferiority test. Considering ineligible cases and an approximately 10% dropout rate, the target enrollment number was set at 320 cases.

### 2.11. Significance of the D-CURE Trial

If the efficacy and safety of UEMR are proven in this RCT, it is expected to be recognized as the standard treatment for SNADETs ≤ 20 mm. Owing to the absence of submucosal injection, UEMR is a simpler and more cost-effective technique compared to CEMR and is anticipated to become the primary method of EMR.

### 2.12. Data Collection and Management

After obtaining written consent from the patients, the investigators will reconfirm that the cases meet all inclusion criteria and do not meet any exclusion criteria. Subsequently, investigators who have been pre-registered in the D-CURE Electronic Data Capture (EDC) system will enter anonymized data into the web-based EDC system of the UMIN Internet Data and Information Center for Medical Research (UMIN INDICE). Changes made in the EDC will be recorded in a log, including information on who made the changes and when. The EDC system and associated databases will be secured and protected with specific passwords. The collected data will be stored online. However, if patients request data deletion after withdrawing their consent, their data will be deleted.

### 2.13. Planned Statistical Analyses

In this trial, the primary analysis will be conducted for the primary endpoint, the 1-year RFS, and this will be the final analysis. The analysis will be conducted within a year from the end date of final data collection of the registered cases by an independent biostatistician separate from the research physicians. Given the short planned registration and observation periods after protocol treatment, an interim analysis aimed at early termination will not be conducted.

The full analysis set (FAS) will consist of all enrolled patients who are randomized to the study arm, excluding some patients for the following reasons: violation of the eligibility/exclusion criteria, erroneous enrollment, no available data after randomization (e.g., withdrawal of consent), and failure to undergo EMR for SNADET. Cases in which the treatment protocol is changed or discontinued will be included in the FAS. The per-protocol set (PPS), a subset of the FAS, will exclude FAS cases with the following violations: incomplete protocol treatment and other serious protocol violations (e.g., treatment for SNADET exceeding 20 mm). The safety analysis set will include patients enrolled in this study whose treatment protocol is initiated and whose clinical course is followed for up to 28 days after the EMR. Cases in which protocol treatment has not been initiated will not be included in the safety analysis set.

#### 2.13.1. Analysis of Efficacy

The analysis of efficacy will be based on the FAS as the primary analysis population. A similar analysis will be conducted separately for the PPS. Subgroup analysis will also be performed according to preoperative biopsy status (present vs. absent), tumor length (<10 mm vs. ≥10 mm), and lesion location (bulb vs. other portions), which are allocation adjustment factors. Furthermore, an analysis of cases of untraceability (missing values) will be conducted, considering the inability to perform follow-up EGD.

1. Analysis of primary endpoint

The 1-year RFS, the primary endpoint, will be aggregated for each group, and the intergroup difference and the 95% confidence intervals (CI) will be calculated. The null and alternative hypotheses for the non-inferiority test are as follows:

Null hypothesis: 1-year RFS of UEMR, P1 + Δ ≤ 1-year RFS of CEMR, P2 (UEMR is inferior to CEMR by more than Δ)

Alternative hypothesis: 1-year RFS of UEMR, P1 + Δ > 1-year RFS of CEMR, P2 (UEMR is not inferior to CEMR by more than Δ)

If the null hypothesis is rejected at a one-sided 5% significance level in the non-inferiority test, we will conclude that UEMR is not inferior to CEMR. If non-inferiority in the 1-year RFS is demonstrated in the primary analysis, a test for superiority will be conducted.

2. Analysis of Secondary Endpoints
  a. *En bloc* resection rate, histological complete resection rate, technical success rate, complete mucosal closure rate, and complete mucosal closure rate with standard clips: The rates for the CEMR and UEMR groups, the point estimate of the intergroup differences, and the 95% CI will be calculated.
  b. Total procedural, resection, and mucosal closure times: Summary statistics will be computed for both groups. The Wilcoxon rank-sum test will be used.
  c. One-year duodenum preservation survival rate: The rates for the CEMR and UEMR groups, the point estimate of the intergroup difference, and the 95% CI will be calculated.
  d. Device cost: Summary statistics will be computed for each group, and the Wilcoxon rank-sum test will be used for comparison.

#### 2.13.2. Safety Analysis

Adverse events: The rates for the CEMR and UEMR groups, the point estimate of the intergroup differences, and the 95% CI will be calculated based on the safety analysis set. Fisher’s exact test will be used for intergroup comparisons.

#### 2.13.3. Exploratory Analysis

An analysis considering the untraceable cases (missing values) will be conducted. If follow-up EGD cannot be performed, the events of the 1-year RFS cannot be observed, potentially affecting the primary endpoint. Therefore, sensitivity analysis considering missing values will be conducted for the primary endpoint. Missing cases in both groups will be randomly imputed to match the proportion of the presence or absence of recurrence or all-cause mortality in the population, excluding cases in which follow-up EGD was not performed from the FAS. The 1-year RFS will be aggregated for each group, intergroup differences will be calculated, and the 95% CI for both groups will be determined using the bootstrap method. Additionally, the 1-year RFS will be calculated for the FAS population, excluding cases where follow-up EGD is not performed, with SNADET recurrence or any deaths considered. Intergroup comparisons will be conducted using the same method described above to confirm that the results remain unchanged.

Moreover, post hoc analysis using multivariate analysis may be conducted to adjust for confounding factors, detect interactions, and explore prognostic factors and predictive markers.

#### 2.13.4. Patient and Public Involvement

Neither patients nor the public were involved in planning the study design or leading the clinical trial.

### 2.14. Ethics and Dissemination

The D-CURE trial is being conducted in compliance with the Declaration of Helsinki. This trial was approved by the certified review board of Tohoku University (No.34158; 22 November 2023). Necessary modifications will be made to the protocol after the research group reaches a consensus and will be approved by the certified review board of Tohoku University before implementation. If there are any protocol modifications, they will need to be approved by the certified review board of Tohoku University. The principal investigator or physician in charge of the research will obtain free and voluntary written consent for participation in the research after fully explaining the research to the patients using explanatory documents (Supplemental Material File). Written informed consent will be obtained from all patients who wish to participate in this study. The patients will be informed that they can withdraw their consent at any time. Patient data will be anonymized before entry into the database to protect privacy. The project principal investigators will have access to their own clean dataset. The trial findings will be disseminated in peer-reviewed journals and conference presentations.

## 3. Discussion

ER is widely used for the treatment of epithelial tumors of the digestive tract, and various ER methods have been reported [26,27,28]. Numerous clinical trials have demonstrated their efficacy and safety [29,30]. Although CEMR is commonly performed to ensure an adequate margin from the muscularis propria before resection, it requires a certain level of endoscopic skill and incurs additional time and costs. Furthermore, drawbacks, such as hematoma formation owing to submucosal injection and challenges in resection caused by the non-lifting of lesions, have been encountered. To address these shortcomings, UEMR, which eliminates the need for submucosal injections, has been developed [11]. Several reports indicate that UEMR can help achieve a higher *en bloc* resection rate and lower recurrence rate, and is more effective than CEMR, especially for colorectal polyps larger than 10 mm [31,32,33]. However, considering the short-term outcomes in previous reports on SNADETs, demonstrating the superiority of CEMR over UEMR in the duodenum remains challenging [15]. Given that local recurrence is the most important outcome in clinical practice, we planned an RCT to establish the non-inferiority of UEMR to CEMR in terms of the local recurrence rate. UEMR not only offers a simpler technique but also does not require specialized devices. Based on the results of the D-CURE trial, widespread adoption of UEMR for the ER of SNADETs is anticipated, along with the dissemination of UEMR techniques.

A recent prospective observational study was conducted in Japan to evaluate the treatment of SNADETs ≤ 20 mm [12]. This study included 155 patients who underwent UEMR from multiple centers. The *en bloc* and histological complete resection rates were 89.8% and 66.9%, respectively, with a 1-year non-recurrence rate of 97.2%. However, our D-CURE trial has several strengths compared to previous studies. First, this is the first RCT conducted across 40 institutions in Japan, enabling the demonstration of safety and efficacy through random allocation and comparison, thus mitigating the possibility of selection bias observed in single-arm prospective trials. We aim to establish the efficacy, safety, and cost-effectiveness of UEMR based on robust results from the D-CURE trial. Additionally, the D-CURE trial includes mucosal closure as a component of the treatment protocol following resection. Mucosal closure has been shown to significantly reduce postoperative complications such as delayed bleeding and perforation by protecting the submucosal and muscular layers from bile and pancreatic juice. Although mucosal closure is considered essential, it is effective only when the ulcer bed is completely covered by the mucosa [34,35]. Although various closure methods using suturing devices have been reported, a simple and cost-effective method is preferred. Because the ulcer bed after UEMR tends to be smaller than that after CEMR, owing to the absence of submucosal injection, suturing with standard clips may be easier. Therefore, in this study, we specified a protocol to use standard clips as the primary method for closure. This approach may highlight differences in the complete mucosal closure rate achieved with standard clips between CEMR and UEMR.

In interpreting the results of this trial, the prespecified non-inferiority margin of 5% should be understood within the specific clinical context of endoscopic treatment for superficial non-ampullary duodenal epithelial tumors. Reported recurrence rates after standard endoscopic resection are low, and when recurrence occurs, it is typically local and amenable to repeat ER. Therefore, a limited increase in recurrence risk does not necessarily translate into irreversible harm or deterioration in long-term prognosis. From this perspective, the selected margin represents a clinically acceptable trade-off when balanced against the potential advantages of UEMR, including procedural simplicity. In addition, although a one-sided significance level of 0.05 was adopted to ensure statistical feasibility in this rare disease setting with an expected low event rate, the findings should be interpreted with appropriate caution. To enhance the robustness of the conclusions, sensitivity analyses using more stringent non-inferiority margins will be conducted, and effect estimates with confidence intervals will be emphasized rather than reliance on a single *p*-value threshold.

One of the limitations of our study is that it is an open-label study. Blinding endoscopists is not feasible during an endoscopic intervention. However, the primary endpoint, 1-year RFS, is not influenced by the endoscopist’s judgment. The inclusion of death from any cause in the primary endpoint may be considered a stringent outcome measure for a study primarily focused on local recurrence after endoscopic intervention. However, given the minimally invasive nature of the procedure and the expected low mortality in this study population, the primary endpoint is anticipated to largely reflect recurrence events rather than mortality. In addition to UEMR, there are methods that use GEL instead of water and partial injection EMR [36], which involves localized injections. However, owing to additional costs and the effort required to perform localized injections, these techniques have not become as widely adopted as UEMR. Furthermore, applying ESD or more advanced-full thickness resection to lesions under 20 mm that can be completely resected with a snare is not considered optimal owing to high complication rates and technical difficulty [15]. Therefore, UEMR was selected for the study arm.

The D-CURE trial will provide crucial information for decision-making regarding the treatment of SNADETs ≤ 20 mm. We anticipate that the most suitable ER method for patients with gradually increasing SNADETs will be firmly established based on solid evidence from the D-CURE trial.

### Trial Status

The study protocol was approved by the certified review board of Tohoku University (no. 34158; 22 November 2023). The trial was registered in the University Hospital Medical Information Network (UMIN) Clinical Trials Registry (UMIN000052778; 17 November 2023). The first patient was randomized on 18 December 2023. The study is expected to end by 30 November 2027.

## Data Availability

No new data were created or analyzed in this study. Data sharing is not applicable to this article.

## Supplementary Materials

The following supporting information can be downloaded at: https://www.mdpi.com/article/10.3390/mps9010030/s1.

## Abbreviations

Underwater endoscopic mucosal resection (UEMR), sporadic non-ampullary duodenal epithelial tumors (SNADETs), conventional endoscopic mucosal resection (CEMR), randomized controlled trial (RCT), endoscopic resection (ER), endoscopic submucosal dissection (ESD), Standard Protocol Items: Recommendations for Interventional Trials (SPIRIT), University Hospital Medical Information Network (UMIN), over-the-scope clip (OTSC), esophagogastroduodenoscopy (EGD), recurrence-free survival rate (RFS), Electronic Data Capture (EDC), UMIN Internet Data and Information Center for Medical Research (UMIN INDICE), full analysis set (FAS), per-protocol set (PPS), confidence intervals (CI).
