# Supplementary material for: Conventional Versus Underwater Endoscopic Mucosal Resection for Superficial Non-Ampullary Duodenal Epithelial Tumors ≤ 20 mm: Study Protocol for a Multicenter Randomized Controlled Trial (D-CURE Trial)
Source: Methods Protoc. 2026 Feb 23;9(1):30. doi: 10.3390/mps9010030 (PMC12942838; doi:10.3390/mps9010030)
Supplement: Supplementary file 1 [file mps-09-00030-s001.zip › mps-4077082-supplementary.pdf]

Informed consent form

“Conventional versus underwater endoscopic mucosal resection for superficial non-ampullary duodenal epithelial tumors ≤20 mm: study protocol for a multicenter randomized controlled trial (D-CURE trial)“

Introduction

Please read this explanatory document carefully and decide whether or not to participate in the study based on your own free will after giving it sufficient consideration. If you choose not to consent to participate, it will not affect your subsequent treatment in any way. Additionally, even if you decide to participate in the study, you may withdraw at any time if you wish to stop.

Taking the above into account, if you have any unclear terms or expressions, or if you have any questions or concerns, please do not hesitate to ask.

1. About Medical Research

Advancements in the diagnosis and treatment of diseases have been made possible through various research efforts up to the present day. Some research aimed at improving diagnostic and treatment methods requires the participation of patients or healthy individuals. Such research, involving patients or healthy individuals, is referred to as "medical research." The medical research described here has been thoroughly reviewed and approved by an ethics committee* to ensure that participating patients do not suffer any disadvantages, and it has been authorized for implementation by the head of the research institution.

*Ethics Committee: This committee, consisting of both experts and non-experts, reviews research from scientific and ethical perspectives to ensure the safety of patients and healthy individuals. Information such as the ethics committee's procedures, committee member roster, and meeting minutes can be found on the website below for your reference.

Name: Tohoku University Hospital Clinical Research Ethics Committee

Establishment: Tohoku University, a National University Corporation, Head of Tohoku University Hospital

Location: 1-1, Seiryocho, Aoba-ku, Sendai, Miyagi, Japan

URL：https://www.rinri.med.tohoku.ac.jp/portal/

2. Background of This Study

Non-papillary duodenal epithelial tumors are tumors that originate from the mucosa of the duodenum, excluding the papilla. Compared to tumors of the stomach or colon, these tumors are relatively rare. Reports indicate a global trend of increasing incidence for non-papillary duodenal epithelial tumors. Traditionally, surgical operations, which are physically invasive, have been performed for these tumors. However, with the advancement of endoscopic technology, endoscopic resection has become a viable alternative.

For non-papillary duodenal epithelial tumors ≤ 20 mm in size, the standard procedure has been conventional endoscopic mucosal resection (EMR), which involves injecting a liquid into the submucosa (local injection). However, there are instances where local injection is difficult or the endoscopic tools used for EMR are not effective due to the liquid injection. Recently, a new technique called underwater EMR, which does not require local injection but instead fills the duodenum with liquid, has been reported. This underwater EMR is said to offer a simpler method for endoscopic resection. However, there have been no clinical trials directly comparing the efficacy and safety of underwater EMR with conventional EMR, and it is not fully understood whether underwater EMR can achieve comparable therapeutic effects.

Understanding these aspects is expected to lead to the development of improved treatment methods for non-papillary duodenal epithelial tumors.

3. Treatment Methods in This Clinical Trial

In this clinical trial, you will receive either conventional EMR (Group A), which involves local injection for resection, or underwater EMR (Group B). Detailed explanations about the EMR methods and any examinations prior to the EMR will be provided to you by your attending physician.

Group A: conventional EMR (figure 1)

Using an endoscope, a local injection is administered beneath the mucosa of the duodenum to elevate the lesion. The elevated area is then constricted with a metal loop called a snare, and an electric current is applied to resect the lesion along with the elevated portion. The duration of endoscopic resection varies significantly depending on the location and size of the lesion, but it typically takes around 1 to 2 hours.

If, during the procedure, it becomes evident that resecting the lesion is more difficult than anticipated or if complications arise that require a change in the treatment approach, the conventional EMR procedure may be discontinued. In such cases, the attending physician will switch to a treatment method deemed appropriate.

Depending on the facility, the usual hospitalization period after endoscopic treatment is approximately one week. Additionally, in this clinical trial, the conventional EMR will be performed by physicians who have experience with at least 20 cases of conventional EMR.

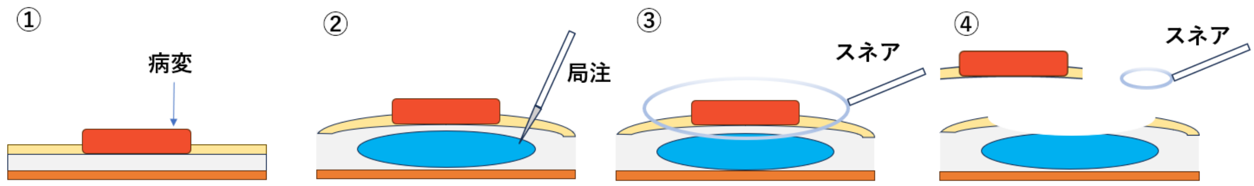

Group B: underwater EMR (figure 2)

Using an endoscope, the duodenal lumen is filled with liquid. As the lumen is filled with liquid, the tension of the duodenal mucosa is relieved, causing the lesion to float upward within the lumen. The elevated lesion is then constricted with a snare and resected using electric current. The duration of endoscopic resection varies depending on the location and size of the lesion, but it typically takes around 1 to 2 hours.

In underwater EMR, if it becomes evident during the procedure that resecting the lesion is more difficult than expected, or if complications arise that suggest a change in the treatment approach is necessary, the underwater EMR may be discontinued. The attending physician will then switch to a treatment method deemed appropriate.

Depending on the facility, the hospitalization period following underwater EMR is generally around one week, similar to the period following conventional EMR. Additionally, in this clinical trial, underwater EMR will be performed by physicians who have experience with at least 20 cases of underwater EMR.

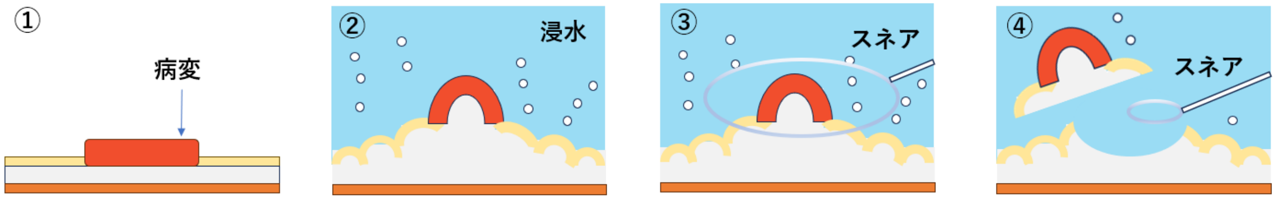

4. Complications

Below are explanations of complications associated with endoscopic resection, listed in order of frequency. While some complications can be anticipated to a certain extent, individual differences mean that not all complications can be predicted completely. Not all of the listed complications will necessarily occur. Additionally, although there is no high-quality data directly comparing conventional EMR and underwater EMR, the incidence of adverse events is considered to be similar between the two methods.

1. Intraoperative Bleeding

   Bleeding from the resection site may occur during endoscopic treatment. Most cases can be managed promptly with endoscopic hemostasis, but in rare instances, blood transfusion or additional surgery may be necessary.

2. Intraoperative Perforation

   The snare used in endoscopic treatment may damage the muscular layer of the duodenum, potentially causing a perforation. This is estimated to occur in about 0.5% to 1% of cases. Most cases improve with endoscopic or conservative treatment, but emergency surgery may be required in some situations.

3. Delayed Bleeding

   Bleeding from the resection site can occur after the endoscopic treatment, either during hospitalization or after discharge, in about 2% to 3% of cases. Bleeding can occur up to two weeks after the procedure. Most cases can be managed with endoscopic hemostasis, but in rare cases, blood transfusion or surgery may be needed.

4. Delayed Perforation

   Damage to the resection site from bile or pancreatic fluid can cause perforation of the duodenum even after the endoscopic procedure has been completed successfully. This is reported to occur in about 0.2% of cases, and if conservative treatment is not effective, surgery may be required.

5. Aspiration Pneumonia

   During or after the endoscopic treatment, aspiration of saliva or gastric fluid may lead to pneumonia. Treatment typically involves antibiotics, but in some cases, mechanical ventilation

may be necessary.

6. Death

   Summary reports from other facilities indicate that the incidence of death related to endoscopic treatment (treatment-related mortality) was 0% in the short term. However, theoretically, treatment-related mortality is a possible outcome.

5. Study Content and Duration

1）Purpose of the Study

The purpose of this study is to investigate the efficacy and safety of underwater EMR by comparing it with conventional EMR (Group A), which involves local injection for resection, in patients with non-papillary duodenal epithelial tumors ≤ 20 mm.

This study is a multi-center collaborative research project with Tohoku University Hospital serving as the coordinating institution.

2）Criteria for Participation in the Study (Reasons for Selection as a Candidate)

You may participate in this study if you meet the following criteria. However, please be aware that even if you provide consent, you may not be able to participate based on the results of subsequent examinations.

You are eligible to participate in this study if you meet the following criteria:

1. Diagnosis of Adenoma or Adenocarcinoma: Diagnosed with adenoma or adenocarcinoma via endoscopic examination or endoscopic biopsy.
2. Lesion Size: The lesion is diagnosed as being 20 mm or less in its longest diameter by an endoscopic examination prior to treatment.
3. Depth of Invasion: The primary lesion is diagnosed as having mucosal invasion only, as determined by pre-treatment endoscopic examination.
4. Location of the Lesion: The lesion is located in the bulb, descending, or horizontal part of the duodenum and does not show continuity with the papilla.
5. Non-Stalked Lesion: The lesion is not pedunculated.

6. Single Lesion: Planning to undergo endoscopic treatment for a single lesion.
7. No Prior Surgery: No history of gastric or duodenal resection.
8. Age: Aged between 18 and 85 years at the time of registration.
9. Functional Status: Able to walk, manage personal care, and spend more than 50% of the daytime outside of bed.
10. Informed Consent: Capable of providing written consent for participation in the study.

However, individuals who meet the following criteria are not eligible to participate in this study:

1. Familial Adenomatous Polyposis: Diagnosed with familial adenomatous polyposis.
2. Systemic Infection: Have a systemic infection requiring treatment.
3. Pregnancy or Breastfeeding: Currently pregnant or breastfeeding.
4. Mental Health Issues: Have psychiatric disorders, mental symptoms, or dementia that make participation in the study difficult.
5. Unstable Angina or Recent Myocardial Infarction: Have unstable angina (angina that has developed or worsened in the past 3 weeks) or a history of myocardial infarction within the past 6 months.
6. Antithrombotic Medication: Taking multiple antithrombotic medications for which endoscopy is advised to be postponed according to gastrointestinal endoscopy guidelines.
7. Respiratory Disease: Have a respiratory condition that requires continuous oxygen supplementation.
8. Short Life Expectancy: Expected to have less than one year of life expectancy.
9. Other Reasons: Any other conditions deemed inappropriate for participation by the study physician.

3）Planned Duration and Expected Number of Participants

This study is scheduled to be conducted from the date of implementation approval by the head of the medical institution until approximately November 2027. We plan to enroll 160 patients in Group A, 160 patients in Group B, for a total of 320 participants.

4）Study Methods and Observation/Examination Schedule

If you consent to participate in this clinical trial, your attending physician will register you for the study, and treatment will begin.

After registration, you will be randomly assigned to receive either conventional EMR (Group A) or underwater EMR (Group B) with a 50/50 probability. This randomization process ensures that neither you nor your attending physician will influence the choice of treatment. Allowing personal choice could lead to biased results due to differences in patient characteristics between the treatment groups, which would affect the validity of the clinical trial outcomes. Randomization is considered the best method for comparing treatments when the effectiveness of each treatment is not yet known and is widely used in clinical trials worldwide.

You will undergo an endoscopic examination.

During hospitalization, your recovery and overall condition will be monitored, including checking for complications and assessing your health status through blood tests and monitoring of fever.

You will undergo an endoscopic examination 12 months after the EMR procedure. During this 12-month follow-up endoscopy, biopsies will be taken to confirm that no residual lesions remain.

The number of examinations conducted while participating in this clinical trial will be the same as if you received the same treatment outside of the trial. The total duration required for your participation in this study, including the 1-year follow-up period after the endoscopic treatment, will be approximately 12 months from the time you give your consent.

5）Treatment After Study Completion

After the treatment conducted as part of this clinical trial is completed, we will suggest the best possible treatment options based on your condition at that time.

6）Expected Benefits and Risks/ Burdens of Participation

Expected Benefits

The conventional EMR and underwater EMR treatments used in this study are standard procedures

covered by health insurance. As all treatment costs during the study period will be covered by your insurance and out-of-pocket expenses, there will be no direct benefit to you from participating in the research.

Risks/Burdens

Both conventional EMR and underwater EMR carry the potential risk of complications, as described in "4. Complications." These complications could have adverse health effects, which are risks that can also occur in routine clinical practice. We have carefully planned this clinical trial to minimize these risks and will make every effort to reduce your risks during the study. The study administration will monitor adverse events associated with EMR to ensure they remain within expected ranges. If severe or unexpected adverse events occur, they will be carefully reviewed and appropriate measures will be taken.

Based on previous research, we expect that underwater EMR will have treatment efficacy comparable to that of conventional EMR. However, depending on the results of the trial, there is a possibility that underwater EMR may perform worse than conventional EMR. In such cases, there could be increased risks of recurrence or other disadvantages compared to conventional EMR.

6. Other Treatment Options if You Do Not Participate in This Study

If you choose not to participate in this study, you can still receive treatment by selecting either conventional EMR or underwater EMR, based on consultation with your physician.

7. Freedom to Participate and Withdrawal of Consent

1. Voluntary Participation

   Participation in this study is entirely voluntary. Please read this explanatory document carefully and discuss it with your family if needed. Decide whether to participate based on your own free will. If you have any questions, feel free to ask. If you agree to participate, please provide your signature or seal on the consent form. If you choose not to participate, it will not affect your future treatment in any way.

2. New Information

   If new information arises during the study that could affect your decision to continue participating, we will inform you promptly. At that time, we will ask you to confirm whether you wish to continue

participating in the study.

3. Right to Withdraw

   You may withdraw from the study at any time. If you decide to stop participating, please inform the study team without hesitation.

4. Impact on Future Treatment

   Choosing not to participate or withdrawing from the study will not result in any disadvantages for your future treatment. We will provide the best available treatment for you at that time.

5. Handling of Data After Withdrawal

   If you withdraw your consent, any samples you provided will be promptly discarded. However, any information collected up to that point may be used for the study in a non-identifiable form. If you do not want any of your information to be used, please inform your physician. Note that if research results have already been published or if data has been fully anonymized at the time of withdrawal, it may not be possible to remove your information from the study.

8. Protection of Personal Information and Handling of Research Results

The study will be conducted with strict protection of your personal information.

1. Data Anonymization and Management

   Information obtained from this study will be anonymized by removing identifiable personal details such as your name and replacing it with a registration number. This anonymized data will be managed so that individuals cannot be identified. The correspondence between registration numbers and patient identities will be securely stored at our institution, ensuring that no personal information is disclosed from the data sent. Results of the study will be published in academic conferences or medical journals, but your name or any identifying information will not be used.

2. Access to Records

   If you participate in the study, the study team (including ethics committee members from this and other institutions) may need to review your records (such as medical charts and endoscopic examination data) to ensure the study is conducted properly. These individuals are bound by confidentiality agreements, so your personal information will be protected.

3. Data Storage and Disposal

   Data will be stored by the collaborating research institutions and our hospital for 5 years from the end of the study or 3 years from the date of result publication, whichever is later. After this period, paper records will be shredded, and electronic records will be rendered unreadable through physical or electronic means before disposal.

   Additionally, data will be stored in anonymized form using the UMIN Internet Medical Research Data Center (INDICE). The collected data will be preserved indefinitely. Information about the location of the UMIN INDICE cloud servers can be found on the following page and will be updated there if changes occur. The data aggregated on the cloud will be analyzed at the Department of Data Science, Kyoto Women's University.

4. Notification of Important Findings

   If the study yields significant findings related to your health, we will inform you of the results.

9. Research Funding and Conflicts of Interest

To ensure fairness, the research group discloses any potential conflicts of interest with external entities in this information document. The funding for this research comes from donations to Tohoku University.

A "conflict of interest" occurs when economic or financial relationships with external parties might compromise, or be perceived to compromise, the fairness or integrity of the research. Currently, there are no conflicts of interest associated with this study. Should any arise in the future, the research will continue under the management of the institution's conflict of interest policies, maintaining fairness and transparency regarding any relationships with external entities.

If any patents or intellectual property rights arise from the results of this research, they will be attributed to the research institution and researchers. They will not be attributed to you.

10. Handling of Health Issues Arising from the Research

1. If Health Issues (Side Effects) Occur: If you experience any new symptoms or notice any changes during the research or after its completion, please inform your attending physician or the research

contact person. Additionally, if you are hospitalized or seek medical care at other departments or institutions due to reasons not related to the research (such as accidents or injuries), please notify us.

2. Treatment and Compensation for Health Issues: If participating in this research directly causes any health issues (such as complications), appropriate treatment will be provided. Medical expenses for this treatment will be covered by your health insurance. However, there will be no financial compensation for any health issues resulting from participation in this research.

11. Conditions Under Which Participation in the Research May Be Terminated

Even if you are participating in the research, it may be discontinued under the following circumstances. If participation is terminated, you may still undergo an examination to check your health status if EMR for non-papillary duodenal epithelial tumors has been performed. Please be aware that, unless you specifically request otherwise, the medical records and test results collected up to that point will be used in relation to this research.

1. If You Decide to Withdraw from the Research: If you express a desire to withdraw from the research, participation will be discontinued.
2. If Your Health Condition Worsens: If your health condition deteriorates and the research team decides to discontinue participation, it will be stopped.
3. If Pregnancy is Confirmed: If it is discovered that you are pregnant, participation will be terminated.
4. If You No Longer Meet Eligibility Criteria: If it is determined that you no longer meet the eligibility criteria for the research, participation will be discontinued.
5. If Disease Progression or Severe Complications Occur: If it is discovered during EMR treatment that the disease has progressed more than initially anticipated, or if severe complications are observed during the endoscopic treatment, and the research team decides that it is better to stop the EMR treatment.
6. If the Entire Study is Discontinued: If the entire research study is halted, your participation will also be discontinued.
7. If the Research Team Decides to Terminate Participation: If the research team determines that it is

better to discontinue your participation for any reason.

12. Possibility of Future Use for Research / Provision to Other Research Institutions

The data you provide may be used for future research. This means that data collected for this clinical trial might be utilized in different research studies beyond this trial. While there are no current plans for this, if important questions arise in the future, the data already collected may be used for further research, subject to approval by the Effectiveness and Safety Evaluation Committee of this study. This could include use in studies conducted domestically or internationally.

For example, the data might be used in combination with data from another clinical trial focusing on non-papillary duodenal epithelial tumors or with data from trials involving similar treatment methods. If there is a need to provide data to other research groups, it will be provided in a form that does not include personally identifiable information.

Additionally, if the data collected in this clinical trial is insufficient for analysis, further investigation may be conducted through your attending physician. Any such additional research will only proceed after obtaining approval from the institution's ethics review board.

13. Costs Related to the Study

Since this research is conducted within the scope of standard medical care, you will be responsible for paying any co-pays related to consultations, tests, medications, and hospital stays according to your health insurance plan. There will be no additional financial burden as a result of participating in this study. No compensation or honorarium is provided.

14. Important Guidelines to Follow

While you are participating in this study, please adhere to the following guidelines:

1. Report Any Health Changes: If you experience any changes in your condition or notice any new symptoms, please contact us immediately.
2. Inform Us About Other Medical Visits: If you visit another hospital or plan to do so, please inform the

study team. With your consent, we may contact your primary doctor to discuss your participation in the study and inquire about your treatment.

3. Notify Us of Schedule Changes: If you are unable to attend your scheduled appointment due to unforeseen circumstances, please contact the study team as soon as possible.
4. Update Your Contact Information: If you change your address or phone number, please inform your primary doctor immediately.
5. Continuing Participation After Transfer: If you need to transfer to another facility after your EMR procedure, we would like you to continue participating in the study. In such cases, we may contact you at home to check your health status or request that your new facility provide your medical information for our records.

15. Information Disclosure and Access to Research Documents

The overview of this study is registered and publicly available in the UMIN-CTR database. Once the study progresses and data is collected, results will also be published in the database, but any information that could identify individuals will not be disclosed.

If you would like to view documents detailing the methods and other aspects of this study, we can provide them to you, excluding sections related to personal information of other participants or any information that might compromise the originality of the research. Please request access from the study team.

16. Contact Information

Rest assured that the best possible treatment will be provided for this condition, beyond the use of the drugs/medical devices involved in this study. If you have any questions or concerns about the research, please contact the consultation office.

(Please note that for research projects not yet specified, we are unable to provide information due to confidentiality and intellectual property protection.)

The overall responsible person for the clinical trial and contact information are as follows:

Principal Investigator:

Waku Hatta

Department of Gastroenterology, Tohoku University Hospital

Masao Yoshida

Division of Endoscopy, Shizuoka Cancer Center

17. Research Organization

This study is a multicenter collaborative research. The participating institutions are as follows:

Institution	Institutional Principal Investigator	
Aichi Cancer Center	Tsutomu Tanaka	
Ishikawa Prefectural Central Hospital	Naohiro Yoshida	Naohiro Yoshida
Ibaraki Prefectural Central Hospital / Ibaraki Prefectural Cancer Center	Hajime Ishibashi	
Iwate Medical University	Takayuki Matsumoto	Yousuke Toya
Osaka Medical and Pharmaceutical University Hospital	Taro Iwatsubo	
Osaka Metropolitan University Hospital	Shusei Fukunaga	
Osaka International Cancer Institute	Satoki Shichijo	
Okayama University Hospital	Yasushi Yamasaki	
Kanagawa Cancer Center	Kohei Takizawa	
Kansai Rosai Hospital	Shinjiro Yamaguchi	
Kitakyushu City Medical Center	Yorinobu Sumida	
Kitasato University School of Medicine	Chika Kusano	Takuya Wada
Kyoto Katsura Hospital	Yasuyuki Tanaka	
Kyoto University Hospital	Mitsuhiro Nikaido	
Kyoto Prefectural University of Medicine Hospital	Osamu Dohi	
Gunma University Hospital	Toshio Uraoka	Yoji Takeuchi
Keio University School of Medicine	Motohiko Kato	Yusaku Takatori
Shinsapporo Hospital	Takenori Aoki	
Kobe City Medical Center General Hospital	Yohei Yabuuchi	
National Cancer Center Hospital	Yutaka Saito	
National Cancer Center East Hospital	Tomonori Yano	Hironori Sunagawa

Shikoku Cancer Center	Aki Hasebe	Norifumi Nishide
Shizuoka Cancer Center	Masao Yoshida	
Shimane Prefectural Central Hospital	Yoichi Miyaoka	Masaki Tanaka
Kawasaki Rinko General Hospital	Ichiro Oda	
Takatsuki Hospital	Hiroaki Sawai	
Chiba Cancer Center	Yoshiyasu Kitagawa	
Tsuyama Central Hospital	Ryuta Takenaka	
Tokyo Metropolitan Bokutoh Hospital	Yohei Furumoto	
Tohoku University Hospital	Waku Hatta	
Toyama Prefectural Central Hospital	Mitsuru Matsuda	
Nagoya City University Hospital	Takaya Shimura	
Nagoya University School of Medicine	Takashi Hirose	
Niigata Cancer Center	Masaaki Kobayashi	
Hiroshima City North Medical Center Asa Citizens Hospital	Shinji Nagata	
Hiroshima City Hiroshima Citizens Hospital	Masahiro Nakagawa	Koji Miyahara
Hiroshima University Hospital	Shirou Oka	Hidenori Tanaka
Hyogo Medical University	Toshihiko Tomita	
Hyogo Cancer Center	Yoshinobu Yamamoto	Noriko Nishikawa
Yokohama City University Medical Center	Kingo Hirasawa	

Consent Form

To: [Hospital Director's Name], Director of [Hospital Name]

"Conventional versus underwater endoscopic mucosal resection for superficial non-ampullary duodenal epithelial tumors ≤20 mm: study protocol for a multicenter randomized controlled trial (D-CURE trial)"

I have received an explanation from the study staff regarding this research and have understood it fully. I will receive a copy of this consent form after signing.

☐ I consent to participate in this study.

Participant

Date of Consent: Year ______ Month ________ Day ______

Contact Information (Phone Number): ______________________

Name: ________________________________ (Signature or Seal)

Consent Explanation Provided By

Date of Consent: Year ______ Month ________ Day ______

Department: ______________________________________

Name: ________________________________ (Signature or Seal)
